# Inactivation of HIV-1 in breast milk by treatment with the alkyl sulfate microbicide sodium dodecyl sulfate (SDS)

**DOI:** 10.1186/1742-4690-2-28

**Published:** 2005-04-29

**Authors:** Sandra Urdaneta, Brian Wigdahl, Elizabeth B Neely, Cheston M Berlin, Cara-Lynne Schengrund, Hung-Mo Lin, Mary K Howett

**Affiliations:** 1 Department of Microbiology and Immunology, Penn State College of Medicine, Hershey, Pennsylvania 17033 USA; 2 Department of Microbiology and Immunology, Institute for Molecular Medicine and Infectious Diseases, Drexel University, College of Medicine, Philadelphia, Pennsylvania 19104 USA; 3 Department of Neural and Behavioral Sciences, Penn State College of Medicine, Hershey, Pennsylvania 17033 USA; 4 Department of Pediatrics, Penn State College of Medicine, Hershey, Pennsylvania 17033 USA; 5 Department of Pharmacology, Penn State College of Medicine, Hershey, Pennsylvania 17033 USA; 6 Department of Biochemistry, Penn State College of Medicine, Hershey, Pennsylvania 17033 USA; 7 Department of Health Evaluation Sciences, Penn State College of Medicine, Hershey, Pennsylvania 17033 USA; 8 Department of Bioscience and Biotechnology, Drexel University, College of Medicine, Philadelphia, Pennsylvania 19104 USA

## Abstract

**Background:**

Reducing transmission of HIV-1 through breast milk is needed to help decrease the burden of pediatric HIV/AIDS in society. We have previously reported that alkyl sulfates (i.e., sodium dodecyl sulfate, SDS) are microbicidal against HIV-1 at low concentrations, are biodegradable, have little/no toxicity and are inexpensive. Therefore, they may be used for treatment of HIV-1 infected breast milk. In this report, human milk was artificially infected by adding to it HIV-1 (cell-free or cell-associated) and treated with ≤1% SDS (≤10 mg/ml). Microbicidal treatment was at 37°C or room temperature for 10 min. SDS removal was performed with a commercially available resin. Infectivity of HIV-1 and HIV-1 load in breast milk were determined after treatment.

**Results:**

SDS (≥0.1%) was virucidal against cell-free and cell-associated HIV-1 in breast milk. SDS could be substantially removed from breast milk, without recovery of viral infectivity. Viral load in artificially infected milk was reduced to undetectable levels after treatment with 0.1% SDS. SDS was virucidal against HIV-1 in human milk and could be removed from breast milk if necessary. Milk was not infectious after SDS removal.

**Conclusion:**

The proposed treatment concentrations are within reported safe limits for ingestion of SDS by children of 1 g/kg/day. Therefore, use of alkyl sulfate microbicides, such as SDS, to treat HIV1-infected breast milk may be a novel alternative to help prevent/reduce transmission of HIV-1 through breastfeeding.

## Background

As proven in developed countries, MTCT of HIV-1 is preventable with highly active antiretroviral therapy combined with total avoidance of breastfeeding. The most widely promoted mode of replacement feeding is the use of infant formula. However, thus far, it has not been applicable in resource-constrained countries, the epicenter of the HIV/AIDS epidemic. In this setting, lack of clean water, absence of financial resources to purchase formula, and cultural stigma represent stumbling blocks for a generalized implementation of this prevention plan. Alternatives to reduce, if not prevent, the risk of transmission of HIV-1 through breast milk are in demand to act in synergy with antiretroviral regimens that prevent *peripartum *transmission of HIV-1. Here we introduce the novel concept of using microbicides to treat HIV-1 infected breast milk to prevent MTCT of HIV-1.

The alkyl sulfate family of microbicides are agents with both surfactant and protein denaturant properties. The prototypic alkyl sulfate, sodium dodecyl sulfate (SDS, C_12_H_26_O_4_SNa, CAS No. 151-21-3), is an anionic surfactant and detergent. SDS is a common ingredient used in the cosmetic and personal care products industry (e.g., toothpastes, shampoos, bubble baths, dishwashing formulations, moisturizing lotions, baby wipes, etc.), and in the laboratory environment as a denaturing agent in gel electrophoresis and other protein solubilization techniques[1,2]. SDS is listed in the Generally Recognized As Safe (GRAS) list of chemicals of the United States Food and Drug Administration (FDA)[3]. Also, the United Nations Environment Programme (UNEP) has classified SDS as "readily biodegradable" and, after extensive toxicological analysis, UNEP concluded that "sodium dodecyl sulfate is of no concern with respect to human health"[2]. According to this report, the Estimated Human Exposure (EHE) level of SDS on a daily basis is 0.158 mg/kg/day and 0.034 mg/kg/day, in children (15 kg of weight) and babies (5 kg) respectively. This includes exposure by means of body lotions and oral intake by means of contaminated water or food and toothpaste. The maximum safe ingested dose for children is estimated to be up to 1.0 g/kg/day[4].

We have previously reported that SDS and related compounds inactivate sexually transmitted viruses including HIV-1, herpes simplex virus type 2 (HSV-2) and human papillomaviruses [5-9]. SDS can inactivate cell-free macrophage-tropic (i.e., CCR5 receptor-using), T-cell tropic (i.e., CXCR4 receptor-using) or dual receptor tropic HIV-1 (i.e., strain 89.6) with concentrations as low as 0.025%[5,6]. There is an urgent need to develop safer methods to provide infants of HIV-1-infected women the benefits of human milk without the risk of the disease. To this end, the possible use of treatment with alkyl sulfates (i.e., SDS) of breast milk infected with HIV-1 has been examined. We hypothesize that treatment of expressed breast milk with this microbicide will effectively inactivate HIV-1 in breast milk. Efficiency of viral inactivation in breast milk is hereon reported. The effects of microbicidal treatment on breast milk components have also been studied (i.e., gross protein content, immunoglobulins, lipids and energy content, cellular fraction, electrolytes) and no significant changes were observed[10,11]. The results of the biochemical analysis of breast milk treated with SDS will be published elsewhere.

## Results

### Virucidal activity of SDS against HIV-1 in breast milk

**Figure 1:**
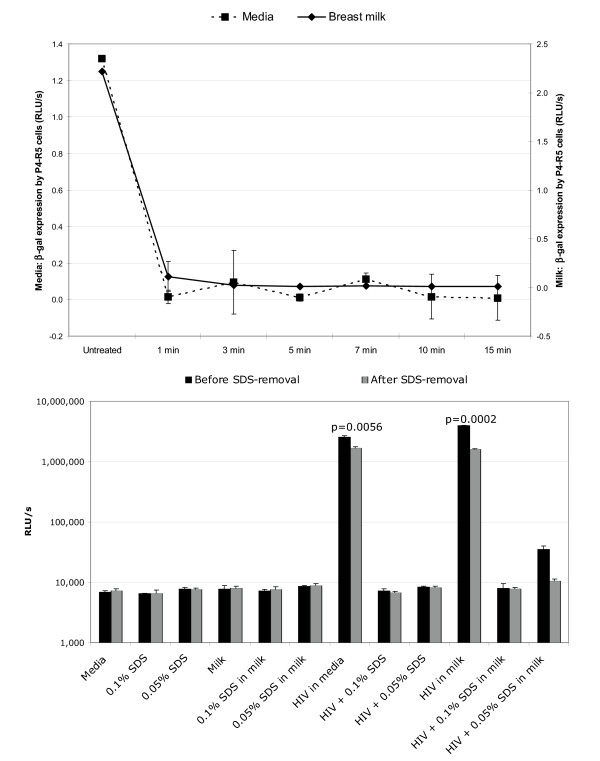
**Irreversible inactivation of cell-free HIV-1 in breast milk treated with SDS. A. **Breast milk from a healthy donor was artificially infected with cell-free HIV-1 IIIB and treated with 0.1% SDS for up to15 min at 37°C prior to plating on P4-R5 MAGI indicator cells (see methods section for details). Two days later, β-gal expression was measured in relative luminescent units per second (RLU/s) in triplicate samples. Results shown are representative of three experiments. **B. **Infectivity of cell-free HIV-1 in breast milk treated with SDS (0.05% and 0.1%) was assessed before and after removal of SDS with SDS-300 Detergent-Out™ (see methods section for details). Results are representative of two experiments, each with triplicate samples.

**Figure 2:**
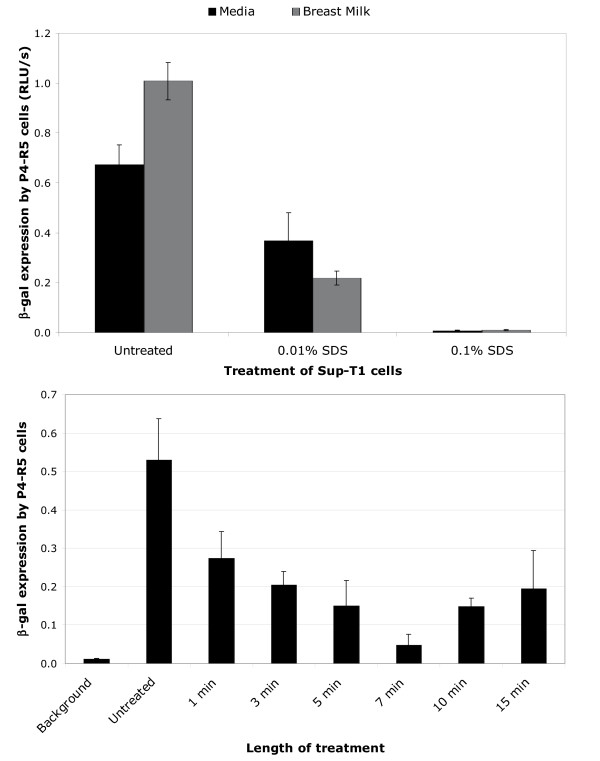
**Inactivation of cell-associated HIV-1 in breast milk with SDS. A. **Supt-T1 cells infected with HIV-1 IIIB were mixed into breast milk from a healthy donor and treated with 1% or 0.1% SDS for 10 min at 37°C prior to plating on P4-R5 MAGI indicator cells (see methods section for details). Two days later, β-gal expression was measured in relative luminescent units per second (RLU/s) in triplicate samples. Levels of β-gal expression by P4-R5 cells correlates with infectivity of cell-associated HIV-1 (i.e., infected Sup-T1 cells). Results are representative of four experiments. **B. **Representative results of the time-course of inactivation of cell-associated HIV-1. Sup-T1 cells in media infected with HIV-1 IIIB were treated for up to 15 min with 0.01% SDS and assayed for infectivity using P4-R5 indicator cells. Samples were assayed in triplicate.

**Figure 3:**
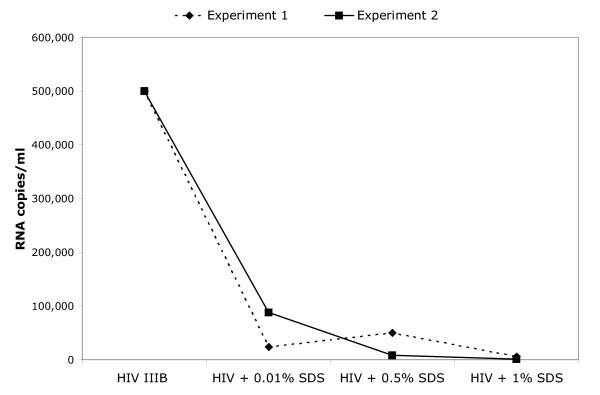
**Reduction of HIV-1 RNA levels in breast milk treated with SDS. **Cell-free HIV-1 IIIB was added to breast milk and treated with ≤1% SDS for 10 min prior to viral load determination using branched DNA technology. Shown are results of 2 independent experiments. Assay sensitivity range: 75–500,000 RNA copies/ml.

The virucidal activity of SDS against cell-free HIV-1 in breast milk was assessed by adding high titer HIV-1 IIIB to breast milk obtained from apparently healthy donors of unknown HIV serostatus. Within 1 min of incubation of breast milk containing cell-free HIV-1 with 0.1% SDS, HIV-1 infectivity was decreased to uninfected control levels (Figure 1A). The minimum concentration of 0.05% was required to observe inactivation of HIV-1 (Figure 1B). Infectivity of cell-associated HIV-1 (i.e., HIV-1-infected Sup-T1 cells) was abolished with treatment with 0.1% SDS. This inactivation was due to induced lysis of Sup-T1 cells at this concentration (data not shown). Cell-associated HIV-1 was partially susceptible to 0.01% SDS (Figure 2A). Nonetheless, even when cell lysis is absent is not an issue low SDS concentrations abolished cell-associated HIV-1 infectivity. With 0.01% SDS, maximum inactivation of infectious cell-associated HIV-1 was achieved within 7 min of treatment (Figure 2B). Using branched DNA technology to determine HIV-1 load in spiked breast milk samples treated with ≥1% SDS, it was determined that viral RNA titers were reduced to undetectable levels (Figure 3).

### Removal of SDS from breast milk

**Figure 4:**
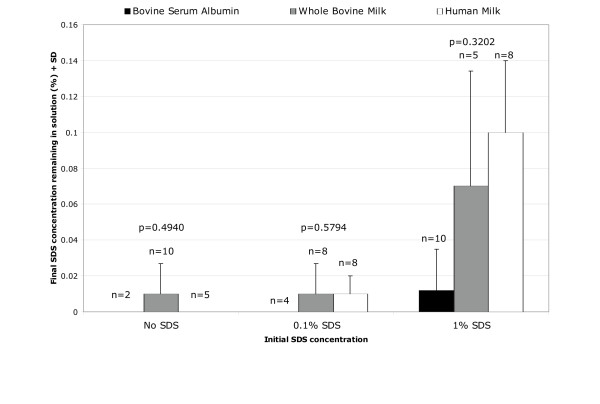
**Efficiency of SDS removal from breast milk, whole bovine milk and bovine serum albumin. **Mixtures of human milk, cow's milk or bovine serum albumin (BSA) containing SDS (0.1%-1%) were subject to SDS removal with SDS-300 Detergent-Out^®^, as per manufacturer's instructions. SDS remaining in solution was quantified spectrophotometrically with the reagents included in the SDS-300 Detergent-Out^® ^kit.

Despite the overall benign nature of SDS, the possibility of removing SDS from breast milk in case it was deemed necessary or desirable prior to feeding was still examined. Several methods were assessed with respect to their efficiency of removing SDS from the breast milk preparations (i.e. potassium salts, Microcon^® ^YM-10 [Amicon^®^, Inc.], SDS 300-Detergent-Out^® ^[Geno Technology, Inc.]). Of these, the SDS-300 Detergent-Out^® ^Medi kit was as efficient as potassium salts[12] with respect to the removal of the surfactant from breast milk (data not shown). The mechanism of action of this resin is proprietary information. However, >90% of the SDS initially present was removed from all samples, with a remaining concentration of SDS of 0.1% or less, as determined with reagents provided in the kit (Figure 4). Differences among treatment groups were not statistically significant (p > 0.05). If removal of the microbicide would be necessary or desirable prior to feeding the mother's milk, it is relevant to determine the potential reversal of the antiviral effect after removal of SDS. To this end, the effect of SDS removal with Detergent-Out™ on infectivity of HIV-1 was also assessed. HIV-1 infectivity was not recovered either after removal of SDS (Figure 1B). Passage of virus solutions through the resin itself decreased infectivity by 40% – 60% (Figure 1B). Paired t-test of HIV-1-infected media and milk samples before and after being passed through the column showed this difference to be statistically significant (Media p = 0.0056, Milk p = 0.0002).

## Discussion

We have previously shown that SDS, has broad-spectrum microbicidal activity, including anti-HIV-1 activity with concentrations as low as 0.025% [5-9]. The positive impact of feeding mother's own milk on infant health and survival are well known and promoted, even in the context of HIV-1 infection [13-15]. Here we report that, with concentrations as low as 0.1% SDS (1 mg/ml), we can inactivate *in vitro *high titers of HIV-1 added to breast milk. This is evidenced by the irreversible loss of infectivity of cell-free and cell-associated HIV-1, and by significant decrease in HIV-1 RNA titers. At treatment concentrations of 0.1% SDS, Sup-T1 cells were lysed contributing to the lack of infectivity observed. This result is congruent with our previously reported findings[16]. However, T cells, as well as macrophages, in colostrum were conserved after treatment with this concentration (data not shown). This discrepancy is possibly due to differences in membrane lipid and protein composition among these cell populations[17]. At this time, we do not understand why the efficiency of treatment with 0.01% SDS in inactivating cell-associated HIV-1 in breast milk is lower at 10 and 15 min of treatment. However, this should not be confused with increased infectivity because infectivity at these time points was still significantly reduced relative to the untreated milk sample (Figure 2B).

Adequate methods of milk storage were put in place to minimize the effects of freeze-thaw cycles on milk components[18,19]. Surprisingly, P4-R5 cells exposed to infected breast milk had higher expression of β-gal than those exposed to infected media (Figures 1A and 2A), and the opposite would have been expected considering the anti-HIV-1 properties inherent to breast milk. However, because the results are expressed in relative luminescent units per seconds (RLU/s), any change in β-gal expression is relative to its matched controlled. Any interference in the milk control would be the same across all milk samples in that experiment because the milk from the same donor was used for all test samples in a single experiment. In addition, we did not pool donors' milk. Therefore, the results and their interpretation should not be affected. When comparing media with breast milk, we are comparing the overall efficacy of SDS in each milieu, and we can observe that efficacy is comparable.

The decrease in HIV-1 RNA titers after microbicidal treatment (Figure 3) has also been observed by other researchers using microbicidal compounds (e.g., Nonoxynol-9) in cervico-vaginal fluids, and may be due to exposure of the viral RNA to RNases in the milk after dissolution of the viral envelope (Deborah J. Anderson, Ph.D., personal communication 12/19/03). If deemed necessary or desirable, a commercially available resin resuspended in water that can remove SDS from milk has been identified. The effects of SDS-removal with this method on human milk nutrients are data presented in a separate manuscript to be published elsewhere, where we report conservation of total milk protein species, conservation of milk immunoglobulins (number and function), and conservation of milk's energy value[10,11].

To date, we have only tested this method on very small volumes (up to 1 ml) using a column device to filter the SDS out of milk. On a greater scale, we envision a model in which breast milk could be expressed manually or mechanically (depending on the living conditions of the nursing mother) into a recipient container or bottle containing SDS. Due to the fast acting effect of SDS against HIV-1 and other pathogens, milk decontamination would occur as warm milk gets expressed into the container. The broad-spectrum action of SDS could also clear milk of other pathogens (e.g., secondary bacterial contamination) that could potentially contaminate it during expression and handling. If removal of SDS prior to feeding would be required, a filtering device comprised by the ion-exchange resin could be located within the nipple manifold in such a way that milk would be filtered through the resin as it is suctioned out of the bottle. If an infant (assuming 5 kg of weight) ingests about 700 ml of breast milk a day[18], at a treatment concentration of 0.1% this would represent an intake of SDS 0.7 g. If 90% of SDS is removed through filtration of treated milk, the final SDS concentration ingested at the end of the day would be 0.07 g; or 0.7 g if milk is instead treated with 1% SDS. Because the toxicological properties of SDS have been broadly studied in animals and humans without toxic effects even at enormous doses (e.g., 258 g in 38 days to an adult human)[2,20-23], the need for removal of SDS still requires further assessment. The metabolism and degradation pathway of SDS and other alkyl sulfates has also been elucidated in *Pseudomonas*, rats, dogs and humans [24-26]. Sulfatase is known to remove the sulfate, and the carbon chain is then metabolized as a fatty acid. We are currently in the process of identifying other candidate microbicides for potential use to decontaminate breast milk with respect to HIV-1 (unpublished observations). Use of edible compounds that can inactivate HIV-1 in breast milk would circumvent the issue of removing the microbicide prior to feeding treated milk[10,27-30].

Among the advantages of microbicidal treatment of expressed HIV-1-infected milk are that it is rapid, discreet (i.e., can be performed in private, minutes to hours before feeding), of low cost, and able to preserve breast milk's nutritional and protective functions. In light of the susceptibility of HIV-1 to heat[31,32], other research groups have looked into the use of heat treatment of milk to inactivate HIV-1 [33-38]. However, heat can be detrimental to important breast milk constituents[39]. In addition, lack of a readily available source of heat in some areas prevents practical application of this option[40]. Refrigeration of expressed milk would not be a *sine qua non *requirement as milk can sit at room temperature for up to 6–8 hours and still be considered bacteriologically safe[18,34], and SDS also has microbicidal activity at room temperature (~23°C) (data not shown). Limitations of our proposed method may be the need for bottle-feeding in settings where cup feeding may be the norm, and milk expression may represent a two-fold stumbling block for a wide spread use of this method because: (1) of the time it may require to express milk, and (2) of the added cost of the final device if a mechanical milk pumping device would be required. An economic assessment of this milk treatment option has not yet been performed. Feasibility of this preventative option also needs to be determined because we, as others, face one of the worst aspects of this epidemic: stigma of not breastfeeding.

## Conclusion

Here we have introduced the novel concept of using microbicides (e.g., SDS) to treat HIV-1 infected breast milk to prevent MTCT of HIV-1. Characteristics of an ideal microbicide for treatment of breast milk include: (1) efficacy at low doses; (2) low level of toxicity; (3) broad-spectrum microbicidal activity; (4) tasteless and odorless; 5) practical to use; and (6) conservation of milk's nutritional and immunoprotective functions. SDS meets most of these requirements. However, we still need to determine the effects of SDS treatment on milk's physical properties (e.g., taste, smell). We anticipate SDS will have similar efficacy to that here reported in naturally HIV-1 infected milk. It remains to be determined, though, whether conservation of milk cells (infected and non-infected) with elimination of cell-free HIV-1 is sufficient to significantly decrease transmission. It is possible that this may be a simple way to prevent milk-borne transmission of HIV-1, while allowing HIV-1-infected mothers to continue providing the nutritional and immunological benefits of breast milk to their children.

## Methods

### Human milk

Breast milk was obtained, from anonymous healthy donors, of unknown HIV serostatus, and regardless of age or parity. The subjects who donated milk were either mothers of children followed in our Outpatient Clinic or nurses that work in our Pediatric Outpatient Clinic. The study was explained to them, and they signed the consent form. The milk samples used were all mature milk (>2 weeks postpartum) unless otherwise stated. Aliquots of unpooled milk were stored at -70°C in polypropylene tubes, and thawed as needed. Because milk samples were not pooled, at least two different donors were used for each experiment to control for outcomes that could be due to individual differences of each donor. This study was performed under approval of the Institutional Review Board of the M. S. Hershey Medical Center (Protocol# 0628EP).

### Microbicidal treatment with sodium dodecyl sulfate (SDS)

Stock solutions of 10% (100 mg/ml) SDS (Bio-Rad Laboratories) were prepared in sterile water and kept at room temperature for up to two weeks. Volume/volume dilutions in media or breast milk were prepared fresh to obtain concentrations of ≤1%. Treatment of human milk was for 10 min at 37°C with final SDS concentrations of 1%, 0.5% or 0.1%. After treatment, SDS was removed with SDS-300 Detergent-Out™ Medi (Geno Technology, Inc.) as described below. In all experiments untreated, uninfected samples were used as controls.

### Removal of SDS and SDS Detection

SDS removal was accomplished by centrifugation of 1 ml of each sample through ion exchange matrix columns (SDS-300 Detergent-Out™ Medi [Geno Technology, Inc.], Extract Clean™ IC-Ba and Extract Clean™ IC-OH [Alltech Associates, Inc.]). Reagents provided in the SDS-300 Detergent Out kit were used to colorimetrically quantify SDS remaining in solution after removal, in addition to an assay using chloroform and methylene blue as previously described[41]. Results were compared to a standard curve of SDS in deionized water. Standard curves of SDS diluted in water were compared to breast milk and whole bovine milk. At concentrations ≤0.1% SDS, there was no significant difference between absorbance measured in milk samples (human or bovine) or water samples using the SDS-300 Detegent Out™ reagents (data not shown). The chloroform-methylene blue assay has the advantage that milk (bovine or human) does not interfere with the absorbance of the sample at any SDS concentration in the standards (≤2%) and, therefore, was used for the later experiments. Optical density of the samples was measured using a visible light spectrophotometer (Spectronic 20^®^, Bausch & Lomb^®^).

### HIV-1 inactivation *in vitro*

Inactivation of infectious cell-free HIV-1 in human milk was studied by a rapid *in vitro *system that quantifies remaining viral infectivity after microbicidal treatment. This system, designated MAGI (Multinuclear Activation of Galactosidase Indicator) assay[42], is based on the use of indicator P4-R5 MAGI cells. These cells are HeLa cells (immortalized cervical cancer cell line) stably expressing the HIV-1 receptor (CD4) and co-receptors (CXCR4 and CCR5) on the surface, and stably transformed with β-galactosidase (β-gal) under the control of the HIV-1 long terminal repeat (LTR). Thus, as a result of HIV-1 Tat activation of the LTR, cells infected with HIV should express β-gal. P4-R5 MAGI cells (8 × 10^4^; obtained through the AIDS Research and Reference Reagent Program, Division of AIDS, NIAID, NIH: P4-R5 MAGI from Dr. Nathaniel Landau) were seeded overnight in 12-well plates. Concentrated HIV-1 IIIB (5 ml; Advanced Biotechnologies, Inc.; Titer: 10^7.67 ^TCID_50_/ml) was treated with SDS (≤0.1% diluted in media or breast milk) for 10 min at 37°C. Media was then added to each reaction tube (1:100 dilution) and plated in triplicate. After 2 h incubation at 37°C, cells were washed and fresh media (2 ml) was added to each well. β-gal expression was measured 46 h later using a chemiluminescent reporter gene assay system (Galacto-Star™ System, Applied Biosystems). All samples were tested in triplicate.

Inactivation of cell-associated HIV-1 was achieved by treating infected Sup-T1 cells (CD4^+ ^human T cells) with SDS (≤1%) for 10 min at 37°C prior to overlaying on P4-R5 cells. In brief, 3 × 10^6 ^Sup-T1 cells were infected with a 1:10,000 dilution of stock HIV-1 IIIB. Infected cells were subject to centrifugation, resuspended in fresh media, and incubated in the presence or absence of SDS (≤0.1%, 10 min at 37°C), three days later. Infected Sup-T1 cells (1 × 10^6^; incubated in the presence or absence of SDS) were co-incubated with indicator P4-R5 cells (1:100 dilution of the inactivation mixture). After 2h, P4-R5 cells were washed and fed with new media. Chemiluminescent expression of β-gal was measured 46 h later. Inactivation of cell-associated HIV-1 in the breast milk was performed in a similar manner, except that infected Sup-T1 cells were resuspended in breast milk instead of media. All samples were tested in triplicate.

All chemiluminescent data was collected with a Fluorosckan^® ^Ascent FL from Thermolab^® ^Systems, except for data in figure 1B, which was collected with a Zylux Corporation^® ^FB15 luminometer. We have determined that the final concentrations of SDS to which P4-R5 cells are exposed to in these assays are not toxic[6].

### HIV-1 RNA load assay

In 10 μl reactions, HIV-1 IIIB (1 μl of virus stock previously diluted 1:100 in media) was added to breast milk or media, and treated with 1%, 0.5% or 0.1% SDS at 37°C. After 10 min, treatment was blocked by adding 990 μl of cold media. Samples were then immediately processed in the Clinical Laboratories of the M. S. Hershey Medical Center for viral load determination using the branched DNA (bDNA) VERSANT^® ^HIV-1 RNA 3.0 Assay (Bayer Corporation, Inc.). This *in vitro *assay is clinically used to directly quantify HIV-1 RNA in plasma of HIV-1-infected individuals.

### Statistical Analysis

Where indicated, samples were tested in duplicate or triplicates. All experiments were repeated two to four times to ensure reproducibility of results. All results are presented here in the form of averages ± standard deviations or as representative results, as applicable to each case. Paired t-test was used to compare samples before and after removal of SDS. ANOVA was used to compare treatment groups.

## List of Abbreviations

SDS – Sodium Dodecyl Sulfate

HIV-1 – Human Immunodeficiency Virus type 1

AIDS – Acquired Immune Deficiency Syndrome

MTCT – Mother-to-Child Transmission

GRAS – Generally Recognized As Safe

FDA – Food and Drug Administration

UNEP – United Nations Environment Programme

EHE – Estimated Human Exposure

HSV-2 – Herpes Simplex Virus type 2

MAGI – Multinuclear Activation of Galactosidase Indicator

LTR – Long Terminal Repeat

bDNA – Branched DNA

## Competing interests

The funding sources, NIH/NIAID No. PO1 AI37829 (MKH), NRSA Fellowship NIH/NICHD No. F32 HD41346 (SU), and Lancaster First United Methodist Church Scholarship Fund (SU), had no role in the study design; in the collection, analysis and interpretation of the data; in the writing of the report; or in the decision to submit the paper for publication. MKH is inventor in and part owner of the U.S. Patent No. 20030129588 that protects the intellectual property surrounding the use of sodium dodecyl sulfate and related alkyl sulfate compounds as microbicidal agents. MKH also serves as President of Renaissance Scientific, LLC, a virtual biotechnology company founded for the purpose of developing licenses related to this patent and other patents. To date, MKH has not received any remuneration in conjunction with alkyl sulfate-related patents. All other authors have no actual or potential, neither personal nor financial conflict of interest that may inappropriately bias their work and/or statements here presented.

## Authors' contributions

SU contributed to the design of the study, acquisition of data, analysis and interpretation of the data, and drafted the manuscript. BW contributed to the design of the study and in the interpretation of the data. EBN participated in the acquisition of data. CMB obtained the IRB approval for this study and coordinated the collection of breast milk samples. CLS participated in the study design, and supervised some of the technical work. HML contributed with the statistical analysis of the data. MKH conceived the study, supervised the technical work, and contributed to the analysis and interpretation of the data. All authors critically revised the manuscript for intellectual content. All authors approved of the final version of the manuscript to be published.
